# Reactive Oxygen Species-Responsive Ferrocene Nanoparticles Delivering Small Interfering RNA Targeting NOP2/Sun RNA Methyltransferase Family Member 2 for Gastric Cancer Therapy

**DOI:** 10.34133/bmr.0209

**Published:** 2025-05-29

**Authors:** Yunsheng Lu, Yibin Huang, Chenchen Mao, Pengfei Shan, Chenkang Wu, Jiongzhou Zhu, Yujie Lin, Zhongyu Li, Mingdong Lu

**Affiliations:** ^1^ Department of Gastrointestinal Surgery, The Second Affiliated Hospital of Wenzhou Medical University, Wenzhou 325000, China.; ^2^ School of Basic Medical Sciences, Wenzhou Medical University, Wenzhou 325000, China.; ^3^ College of Chemistry and Materials Engineering, Wenzhou University, Wenzhou 325027, China.

## Abstract

Silencing NOP2/Sun RNA methyltransferase family member 2 (NSUN2) effectively inhibits gastric cancer (GC) progression but is limited by RNase degradation, rapid renal clearance, and low uptake. Based on the characteristic high levels of reactive oxygen species (ROS) in the tumor microenvironment, this study designed and synthesized a novel ROS-responsive ferrocene nanoparticle loaded with siNSUN2 (PRPFc@siNSUN2). Under ROS conditions, the nanoparticle disintegrates to release siNSUN2. Characterization by proton nuclear magnetic resonance, transmission electron microscopy, dynamic light scattering, and ultraviolet–visible spectrophotometry revealed that PRPFc@siNSUN2 is spherical, with an average diameter of 88.79 ± 1.14 nm, an encapsulation efficiency of 83.10%, and a drug loading capacity of 13.85%. Moreover, these nanoparticles demonstrated excellent stability and, under hydrogen peroxide conditions, exhibited structural disruption leading to the release of siNSUN2, thereby confirming their high ROS responsiveness. In vitro, PRPFc@siNSUN2 markedly enhanced the inhibition of GC cell proliferation, migration, and invasion, and promoted apoptosis, accompanied by increased intracellular ROS and improved siNSUN2 uptake. In vivo studies further confirmed that PRPFc@siNSUN2 markedly enhanced the therapeutic efficacy of siNSUN2 against GC, while exhibiting low cytotoxicity and good biocompatibility. Overall, our findings indicate that PRPFc@siNSUN2, with its favorable morphology, stability, and ROS-triggered release, substantially improves the anti-GC effects of siNSUN2 by inhibiting GC cell proliferation, migration, and invasion, as well as by promoting apoptosis. These results support NSUN2 as a promising therapeutic target and underscore the potential of PRPFc@siNSUN2 nanoparticles in drug delivery, offering a novel strategy to improve clinical outcomes for GC patients.

## Introduction

Gastric cancer (GC), a highly invasive malignancy, accounts for over 1 million new cases globally annually, resulting in approximately 660,000 deaths and ranking among the top 3 causes of cancer-related mortality worldwide [1]. A study predicts a 62% increase in global new GC cases by 2040, reaching 1.77 million [2], posing a significant threat to global public health. Current clinical treatments for GC primarily include surgery, chemotherapy, radiotherapy, and immunotherapy [3]. Despite these therapies extending overall survival to some extent, the 5-year survival rate for advanced GC patients remains below 30% [4]. Moreover, due to vague early symptoms, rapid progression, and a lack of reliable early diagnostic methods, over 80% of GC cases are diagnosed at an advanced stage [5]. Consequently, many GC patients miss the opportunity for surgical intervention, leading to poor prognosis and increased mortality rates [5]. Given these challenges, there is an urgent need to elucidate the molecular mechanisms underlying GC onset and progression and identify effective targets for personalized and targeted therapies to improve patient prognosis and survival rates.

5-Methylcytosine (m^5^C) methylation is an emerging and critical RNA modification mechanism that plays roles in regulating RNA stability, translational control, and cellular functions such as proliferation and differentiation [6]. Studies indicate altered levels of m^5^C modification in various cancers, correlating with cancer pathogenesis and poor prognosis [7]. The catalytic activity of m^5^C modification is predominantly controlled by enzymes from the NOL1/NOP2/SUN domain (NSUN) family and the DNA methyltransferase DNMT2 [6]. Among these, NOP2/Sun RNA methyltransferase family member 2 (NSUN2), also known as myc-induced SUN domain-containing protein, primarily localizes in the nucleus and is encoded by a gene located on chromosome 5p15.31-33 in humans [8]. NSUN2 catalyzes RNA m^5^C formation, influencing the stability of RNA and protein synthesis, thereby regulating cellular processes including proliferation, differentiation, and senescence [9]. Research highlights NSUN2’s pivotal role in cancer progression, closely associated with poor prognosis [7]. For instance, NSUN2-mediated RNA m^5^C modification promotes cell proliferation, migration, and invasion in various cancers such as breast cancer [10] and gallbladder cancer [11] and correlates with adverse outcomes in head and neck squamous cell carcinoma [12]. Additionally, in GC, NSUN2 is considered an oncogene, exhibiting significantly elevated expression levels in GC tissues and cells, where it suppresses apoptosis and promotes proliferation through m^5^C-dependent inhibition of p57^Kip2^ [13], or activates key genes like ORAI2 to facilitate peritoneal metastasis and colonization [14]. Studies further demonstrate that down-regulating NSUN2 effectively inhibits GC cell proliferation and growth [13]. These findings underscore targeted inhibition of NSUN2 as a promising strategy to restrain GC progression.

Small interfering RNA (siRNA) is a nucleic acid-based therapeutic that selectively silences disease-associated genes through sequence-specific binding, demonstrating potential for treating diseases [15]. Nucleic acid synthesis technologies enable precise, rapid, and cost-effective production of siRNA. Compared to small molecules and antibody drugs, siRNA is not only cost-effective but also highly specific, capable of targeting specific mRNA sequences to precisely block gene expression [16]. Moreover, siRNA therapy is attracting attention due to its short development cycle, broad applicability, and diverse uses [17]. Since the approval of the first siRNA therapy (ONPATTRO) by the U.S. Food and Drug Administration (FDA) in 2018, over 20 siRNA therapies have entered clinical trials [18]. In GC treatment, research has confirmed that siNSUN2 effectively inhibits GC cell proliferation and growth [13]. Despite its relative stability and minimal side effects, siRNA faces challenges such as susceptibility to nuclease degradation, rapid renal clearance, and low cellular uptake, necessitating effective delivery systems to achieve silencing effects [19]. However, delivering siRNA effectively to target areas remains a challenging task. To address this challenge, researchers have explored various delivery strategies, particularly nanocarriers. Nanocarriers provide a protective environment for siRNA, enhancing its stability and facilitating targeted delivery to cancer cells [20]. Furthermore, these nanocarriers can improve cellular uptake, thereby enhancing the overall efficacy of siRNA-based therapies [20]. These results suggest that utilizing nanocarriers for siRNA delivery holds promise for improving treatment outcomes and survival rates in cancer patients.

Stimuli-responsive nanoparticles have garnered significant attention among various nanocarriers due to their ability to respond to specific stimuli, including heat, light, reactive oxygen species (ROS), and enzymes, allowing for precise drug release at target sites [21]. Numerous studies have demonstrated that the levels of ROS produced in inflammatory and tumor areas are significantly higher than those in normal cells [22]. As a result, elevated ROS levels serve as ideal triggers for targeted drug delivery in cancer therapy. ROS-responsive polymers can leverage the oxidative capacity of ROS to alter their own structure or properties, enabling appropriate drug release in targeted areas, thereby improving biocompatibility and enhancing therapeutic efficacy [23]. Based on this characteristic, researchers have conducted in-depth explorations into ROS-responsive drug delivery systems. Throughout the development of such systems, various ROS-responsive functional groups, including boron, chalcogen elements, selenium, thioethers, and ferrocene, have been extensively employed in the design of responsive drug delivery systems [24]. Among these, ferrocene, a hydrophobic organic compound, is widely utilized in the production of polymeric nanoparticles due to its advantages in reversible self-assembly and controlled drug release. Notably, ferrocene can be covalently linked to the polyester backbone through ROS-responsive thioacetal connections. When the particles enter a high-ROS environment, ferrocene is triggered to release, and its electron donor–acceptor-conjugated structure can further induce ROS generation [25]. This leads to a “snowball” effect of self-catalyzed degradation, accelerating drug release. Furthermore, the in vivo safety of ferrocene has been validated through clinical trials [26], although its poor physiological stability poses challenges for clinical applications. Currently, ROS-responsive nanocarriers containing hydrophobic ferrocene fragments and hydrophilic carboxyl groups have been developed to achieve controlled drug release [27]. However, these systems still face limitations related to low polymer solubility and instability of physicochemical properties [28]. To address these challenges, researchers have introduced PEGylation technology, which involves covalently attaching polyethylene glycol (PEG) chains to the surface of nanoparticles. The incorporation of PEG not only effectively reduces protein adsorption but also enhances the stability and circulation time of nanoparticles [29]. Due to its excellent biocompatibility and flexibility, PEG can endow nanoparticles with “stealth” properties and has been shown to co-polymerize with ferrocene, further enhancing the stability and biocompatibility of ferrocene-based nanocarriers [28].

This study designed and synthesized a ferrocene-based ROS-responsive nanoparticle (RPFc) and modified it with methoxyl-polyethylene glycol 2000-carboxyl (mPEG2000-COOH) to create a novel ROS-responsive ferrocene polymer nanoparticle (PRPFc) for the delivery of siNSUN2. Through in vitro and in vivo experiments, the therapeutic efficacy of PRPFc against GC was investigated. The results indicated that the siNSUN2-loaded PRPFc nanoparticles (PRPFc@siNSUN2) exhibited excellent biocompatibility and significantly enhanced the therapeutic effects of siNSUN2 on GC in both experimental settings. These findings suggest that NSUN2 is an effective therapeutic target for GC and that PRPFc@siNSUN2 may represent a promising innovative drug for NSUN2-targeted therapy in GC.

## Materials and Methods

### Chemical reagent

All chemical reagents used in this study were commercially available and did not require further purification. Ferrocenecarboxaldehyde, 3-mercaptopropionic acid, 1,6-dibromohexane, mPEG2000-COOH, 1′,1,3,3′-tetramethylguanidine (TMG), deuterated chloroform, allyl ether polyethylene glycol, trifluoroacetic acid (TFA), and succinic anhydride were purchased from Aladdin (Shanghai, China). Dimethyl sulfoxide (DMSO) was purchased from JINSHAN CHEMICAL REAGENT (Chengdu, China).

### Preparation of PRPFc

#### The synthesis of ferrocenyl thioacetal

Ferrocene-9-carboxaldehyde (21.3 g, 100 mmol) and 3-mercaptopropionic acid (27.04 g, 225.00 mmol) were dissolved in 40 ml of ethyl acetate, to which 3 drops of TFA were added to the solution. The mixture was stirred in the dark at 0 °C for 24 h, followed by filtration to collect the white solid. The solid was washed alternately with water and hexane 3 times. Finally, the resulting white solid (ferrocene-based thioether) was vacuum-dried overnight at 40 °C. ^1^H-NMR (400 MHz, CDCl_3_) 3.71 (d, *J* = 7.9 Hz, 1H), 2.53 (d, *J* = 6.5 Hz, 2H), and 2.68 (d, *J* = 6.9 Hz, 2H).

#### The synthesis of ROS-responsive polymers (RPFc)

The synthesized ferrocenyl thioacetal (1.786 g, 8.75 mmol) and 1,6-dibromohexane (1.22 g, 10 mmol) were dissolved in DMSO (10 g), followed by slow addition of TMG (1.88 g, 16.36 mmol). The mixture was stirred at 40 °C for 12 h. The resulting polymer was precipitated into a large volume of deionized water, collected by filtration. The crude product was dissolved in acetone, transferred to ether for precipitation, and dried under vacuum at 40 °C. This process was repeated 3 times to obtain purified ROS-responsive polymer. ^1^H-NMR (400 MHz, CDCl_3_) δ 4.22 (m, *J* = 7.9 Hz, 2H), 3.67 (d, *J* = 7.9 Hz, 1H), 2.89 (d, *J* = 6.5 Hz, 2H), 2.63 (d, *J* = 6.9 Hz, 2H), 1.63 (t, *J* = 6.5 Hz, 2H), and 1.39 (dd, *J* = 7.2 Hz, 8H).

#### The synthesis of ROS-responsive amphiphilic polymer (PRPFc)

The synthesized ROS-responsive polymer and mPEG2000-COOH were dissolved in DMSO, followed by the addition of TMG to the solution. The mixture was stirred at 40 °C for 12 h, then dialyzed against a large volume of deionized water for 72 h. Finally, the resulting emulsion was freeze-dried to obtain the polymer PRPFc. ^1^H-NMR (400 MHz, CDCl_3_) δ 7.3 (s, *J* = 6.9 Hz, 2H), 4.9 (d, *J* = 7.3 Hz, 4H), 4.22 (m, *J* = 6.5 Hz, 8H), 3.35 (m, *J* = 6.9 Hz, 1H), 2.91 (m, *J* = 6.5 Hz, 2H), 2.62 (m, *J* = 7.2 Hz, 6H), 1.79 (m, *J* = 6.5 Hz, 4H), and 1.55 (m, *J* = 6.5 Hz, 4H).

### Preparation and characterization of PRPFc@siNSUN2

To prepare PRPFc@siNSUN2 nanoparticles, siNSUN2 was added to an emulsion containing PRPFc and stirred on a benchtop shaker for 1 h. The successful formation of PRPFc@siNSUN2 nanoparticles was confirmed through proton nuclear magnetic resonance (^1^H NMR) spectroscopy. The morphological characteristics of both PRPFc and PRPFc@siNSUN2 nanoparticles were examined using transmission electron microscopy (TEM, FEI Talos F200X, USA). Dynamic light scattering (DLS) was employed to determine the particle size of PRPFc@siNSUN2 nanoparticles and to monitor size variations over a 30-day period under storage conditions at 4 °C, 25 °C, and 37 °C, thereby assessing their colloidal stability. Hydrodynamic diameters were measured using a nanoparticle size analyzer (Zetasizer Nano ZS, UK). Furthermore, ultraviolet–visible spectrophotometry was utilized to calculate the encapsulation efficiency (EE) and drug loading (DL) of PRPFc@siNSUN2 nanoparticles. DL was also monitored over 30 days at 4 °C, 25 °C, and 37 °C to evaluate the storage stability of PRPFc@siNSUN2. Encapsulation efficiency and loading efficiency were calculated using the formulas EE = [(*m*2 − *m*1) / *m*2] × 100% and DL = [(*m*2 − *m*1) / m total] × 100%, where *m*1 is the free siRNA content in the supernatant, *m*2 is the total siRNA content, and *m* is the total content of polymer and siRNA.

### In vitro drug release

Take 1 ml of 10 mg/ml PRPFc@siNSUN2 nanoparticles and calculate the siNSUN2 content based on the drug loading capacity. The nanoparticles were then enclosed in dialysis bags and placed in centrifuge tubes containing 10 ml of phosphate-buffered saline (PBS), either with or without the addition of 1.0 mM H_2_O_2_. The samples were incubated at 37 °C under gentle shaking for 48 h. At predetermined time points (0, 1, 2, 4, 6, 8, 12, 18, 24, and 48 h), a specific volume of the release medium was withdrawn, filtered through a membrane, and the supernatant was collected. An equivalent volume of fresh PBS was immediately added to maintain a constant volume of 10 ml. The concentration of siNSUN2 in the supernatant at each time point was measured using high-performance liquid chromatography (HPLC, Agilent 1260, USA). The cumulative release rate was then calculated, and a drug release profile was plotted.

### Cell culture

Human umbilical vein endothelial cells (HUVECs) and human GC cells (AGS) were purchased from iCell Bioscience Inc. (Shanghai, China). Cells were cultured in medium containing 5% fetal bovine serum (FBS), 100 IU/ml penicillin, and 100 μg/ml streptomycin, and incubated at 37 °C in a humidified atmosphere with 5% CO_2_.

### The in vitro cytotoxicity and anticancer activity of PRPFc@siNSUN2

HUVECs and AGS cells were used to evaluate the cytotoxicity and anticancer activity of PRPFc@siNSUN2. Cells were randomly divided into Control, siNSUN2, PRPFc, and PRPFc@siNSUN2 groups, and treated accordingly. The treated HUVECs and AGS cells were seeded into 96-well plates (5,000 cells/well), and 10 μl of CCK-8 solution was added to each well. After incubating at 37 °C with 5% CO_2_ for 2 h, absorbance at 450 nm was measured to assess cell viability for both HUVECs and AGS cells.

Additionally, to evaluate the impact of varying concentrations of PRPFc@siNSUN2 on AGS cell viability, CCK-8 assays were performed. Briefly, AGS cells were seeded into 96-well plates (5,000 cells/well) and incubated in a CO_2_ incubator for 1 day. AGS cells were then treated with different concentrations of PRPFc@siNSUN2 (0, 25, 50, 75, 100, 125, and 150 μg/ml) and further incubated. After removing the culture medium, 10 μl of CCK-8 solution was added to each well. Following a 2-h incubation at 37 °C with 5% CO_2_, absorbance at 450 nm was measured to assess the impact of different concentrations on AGS cell viability.

### Cell apoptosis detection

AGS cells from each treated group were seeded in 6-well plates at a density of 2.0 × 10^5^ cells per well and cultured overnight. Subsequently, cells were treated with 2 ml of different complexes diluted in serum-free DMEM for 6 h. Following trypsin digestion, culture media were removed, and cells were washed twice with PBS. The cells were then resuspended in binding buffer and stained with fluorescein isothiocyanate/propidium iodide staining solution at room temperature for 15 min under light-avoiding conditions. Cell apoptosis was analyzed using flow cytometry within 1 h after staining to determine the apoptotic rate.

### Detection of ROS levels

AGS cells were seeded in RPMI 1640 medium without FBS and treated with 10 μM DCFH-DA at 37 °C for 30 min to assess ROS production following various treatments. After incubation, cells were washed 3 times with PBS and observed under a fluorescence microscope.

### Transwell assay

AGS cell migration and invasion capabilities were evaluated using Transwell assays to assess the impact of PRPFc@siNSUN2. Initially, 0.3 ml of cell suspension (3 × 10^5^ cells/ml) was seeded into the upper chamber of Transwell inserts. For invasion assays, Matrigel (80 μl) was precoated onto the Transwell inserts. Subsequently, 0.7 ml of complete medium containing 10% FBS was added to the lower chamber of the Transwell system. After incubating at 37 °C with 5% CO_2_ for 24 h, nonmigratory or noninvasive cells were removed. Cells that migrated or invaded through the Transwell membrane were fixed and stained with 0.5% crystal violet. Cells were then observed under a microscope and photographed for quantitative analysis.

### Detection of cell uptake of PRPFc@siNSUN2 in vitro

To enhance observation of AGS cell uptake of siNSUN2, Cy5 dye was used to label siNSUN2 and PRPFc@siNSUN2. Specifically, siNSUN2 and PRPFc@siNSUN2 (2 mg) were dissolved in 1 ml of 10 mM Hepes buffer (pH 7.2) containing Cy5 (1 mg) and stirred at room temperature for 4 h. The labeled complexes were washed 3 times with 10 mM Hepes buffer to remove unbound Cy5. Following labeling, siNSUN2-Cy5 and PRPFc@siNSUN2-Cy5 were separately co-incubated with AGS cells for 4 h, washed with PBS, fixed with 4% paraformaldehyde (PFA) solution, washed again with PBS, counterstained with 4,6-diamidino-2-phenylindole, and finally observed using confocal microscopy to assess cellular uptake. Additionally, to further investigate the impact of different co-incubation times or concentrations on AGS cell uptake of PRPFc@siNSUN2 nanoparticles, various concentrations of PRPFc@siNSUN2-Cy5 (0, 10, 20, 40, 80, and 100 μg/ml) were co-incubated with AGS cells for 4 h, or the same concentration of PRPFc@siNSUN2-Cy5 (40 μg/ml) was co-incubated with AGS cells for 0, 1, 2, 4, 8, and 10 h. Subsequent procedures were consistent with the aforementioned protocol.

### Animals

Twenty-six male BALB/c mice aged 6 weeks were purchased from the Animal Experimental Center of Wenzhou Medical University. All animal experimental procedures in this study complied with the guidelines for the care and use of laboratory animals. The research protocol was approved by the Ethics Committee of Wenzhou Medical University (approval no. wydw2024-0323).

### In vivo imaging system imaging of PRPFc@siNSUN2

AGS cells (5 × 10^6^ cells/mouse) were subcutaneously implanted into the right flank of BALB/c mice (*n* = 6) to establish a tumor mouse model. When the tumor volume reached 50 mm^3^, PRPFc@siNSUN2-Cy5 (4 μg siRNA per mouse equivalent) was administered via tail vein injection. Experimental mice were anesthetized with 2% isoflurane before imaging. Fluorescence images were acquired at 0, 1, 3, 6, 12, and 24 h postinjection using the PerkinElmer IVIS (in vivo imaging system) Spectrum system. After euthanasia, tumors and major organs (heart, liver, spleen, lungs, and kidneys) were excised and imaged under the same conditions, and fluorescence intensity in selected regions of interest was quantified using imaging software.

### Antitumor activity of PRPFc@siNSUN2 in vivo

The methods for establishing the tumor mouse model and conducting imaging experiments were similar. When tumors reached approximately 50 mm^3^ in size, 20 BALB/c mice were randomly divided into 4 groups: PBS, siNSUN2, PRPFc, and PRPFc@siNSUN2, with 5 mice per group. Each group of mice were treated via tail vein injection with PBS, siNSUN2, PRPFc (5 mg/kg), and PRPFc@siNSUN2 (equivalent to 4 μg siRNA per mouse). During the experiment, mice were weighed and tumor sizes were measured every 2 days. Tumor volume was calculated using the formula: *V* (mm^3^) = 0.5 × tumor maximum diameter × tumor longest diameter perpendicular diameter^2^. After 2 weeks of injections, all mice were euthanized, and tumor tissues were collected for subsequent experimental analyses.

### Pathological examination

Tumor tissue histopathological changes in mice were evaluated using hematoxylin and eosin (H&E) staining. Tissues were fixed in 4% PFA, dehydrated, embedded, and sectioned into 5-μm paraffin slices. These sections were then stained with H&E. After washing, mounting, and drying, pathological changes in the tumor tissues were observed under a microscope.

### Hematocompatibility study

Using in vitro hemolysis assay to evaluate the hematocompatibility of PRPFc@siNSUN2 [30]. Fresh anticoagulated whole blood from healthy mice was collected and mixed with 3.8% sodium citrate anticoagulant solution, centrifuged, and washed with PBS to obtain red blood cells (RBC). The RBC suspension was diluted 10-fold with PBS buffer (pH 7.4). Then, 0.5 ml of the diluted RBC suspension was mixed with 0.5 ml of PRPFc@siNSUN2 at different concentrations (100, 200, 400, 800, and 1,000 μg/ml). Deionized water served as the positive control, and PBS solution served as the negative control. All samples were incubated at 37 °C for 10 min, centrifuged at 10,000 rpm, and the supernatant was collected to measure absorbance at 540 nm. The hemolysis percentage was calculated using the formula:

$$\begin{array}{c} \;\text{Hemolysis}\;\left(\%\right)=\frac{{\mathrm{OD}}_{\text{sample}}-{\mathrm{OD}}_{\text{negative}}}{{\mathrm{OD}}_{\text{positive}}-{\mathrm{OD}}_{\text{negative}}}\times 100\% \end{array}$$

### Measurement of serum biochemical analysis

After intravenous injection of the drug, blood samples were collected from each group of mice. Serum levels of alanine aminotransferase (ALT), aspartate aminotransferase (AST), urea (UREA), creatinine (CRE), RBC, white blood cells (WBC), hemoglobin (HGB), and platelets (PLT) were measured using an automated chemistry analyzer following the methods described previously.

### Histological analysis

After euthanizing rats from both the PBS and PRPFc@siNSUN2 groups, tissue samples from the heart, lungs, liver, spleen, and kidneys were collected. Histopathological evaluation of tissue sections from the heart, lungs, liver, spleen, and kidneys of rats in the Control and PRPFc@siNSUN2 groups was performed using the H&E staining method.

### Statistical analysis

All experiments were conducted with a minimum of 3 replicates. Data are presented as mean ± standard deviation. Statistical analyses were performed using GraphPad Prism 9 (GraphPad Inc, San Diego, CA, USA). Student’s *t* test was used for comparisons between 2 groups, and one-way analysis of variance was used for comparisons among multiple groups. A *P* value less than 0.05 was considered statistically significant.

## Results and Discussion

### Preparation and characterization of PRPFc@siNSUN2

**Fig. 1.**
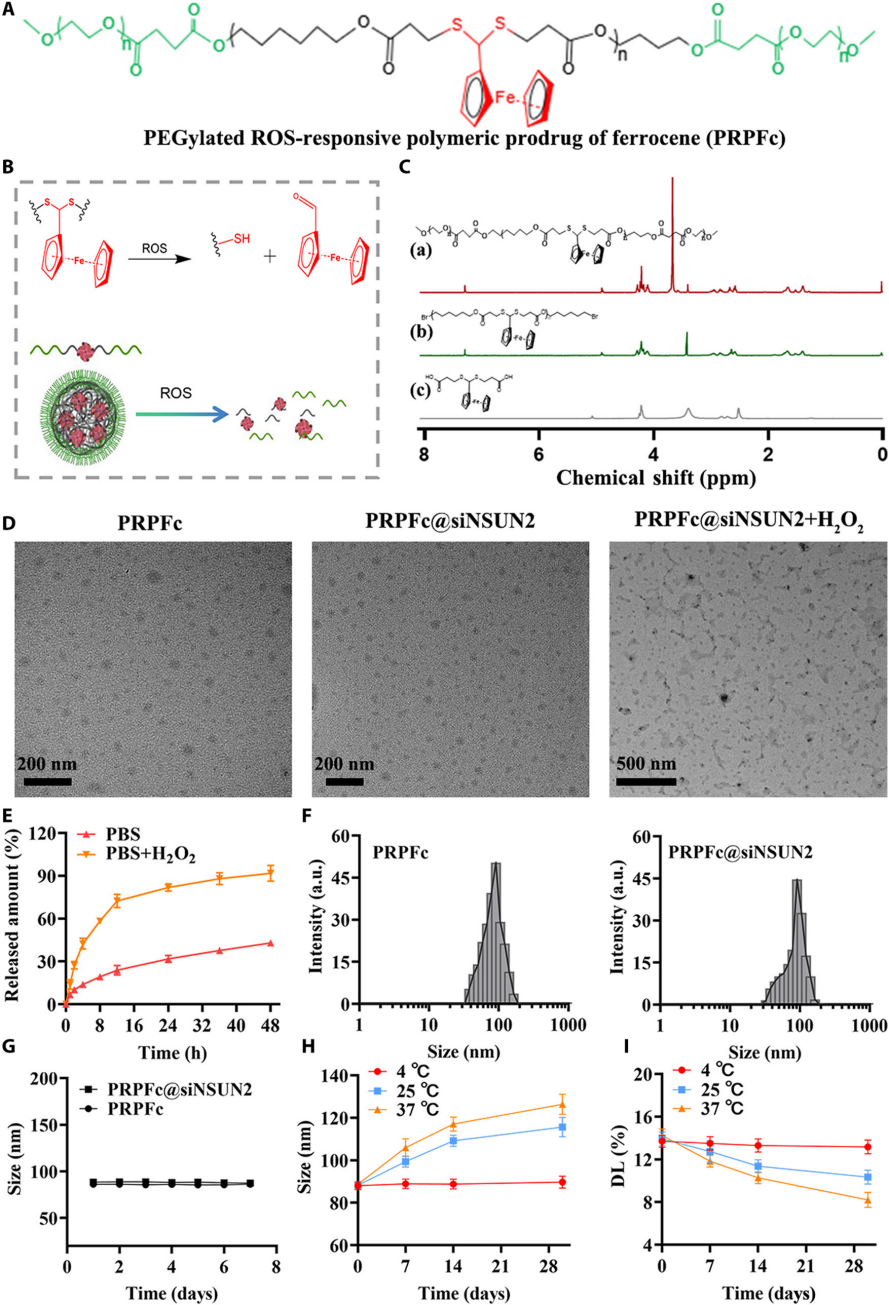
Preparation and characterization of PRPFc@siNSUN2 nanoparticles. (A) Chemical structure of PRPFc. (B) Schematic representation of the ROS-responsive mechanism of PRPFc. (C) ^1^H NMR spectra of PRPFc and intermediate compounds: (a) PRPFc, (b) RPFc, and (c) ferrocenyl thioacetal. (D) TEM images. (E) The in vitro drug release curve of PRPFc@siNSUN2. (F) Particle size distribution of PRPFc and PRPFc@siNSUN2. (G) Particle size stability of PRPFc and PRPFc@siNSUN2 in water over 7 days. (H) The particle size changes of PRPFc@siNSUN2 nanoparticles after being stored at different temperatures (4 °C, 25 °C, and 37 °C) for 30 days. (I) The drug loading changes of PRPFc@siNSUN2 nanoparticles after being stored at different temperatures (4 °C, 25 °C, and 37 °C) for 30 days. DL, drug loading.

As shown in Fig. 1A and B, this study designed and synthesized a novel ROS-responsive nanoparticle drug delivery system (PRPFc), which can effectively load siNSUN2 (PRPFc@siNSUN2). PRPFc nanoparticles covalently bind ferrocene to a biodegradable polyester through a ROS-cleavable thioacetal linker, and the thioacetal part has been demonstrated to be ROS-responsive [31]. The detailed synthetic route is shown in Fig. S1. The intermediates and final products of PRPFc nanoparticles were characterized using ^1^H NMR. The results (Fig. 1C) show that the ^1^H NMR spectrum clearly presents the characteristic proton signals of PRPFc, RPFc, and ferrocene-based thioacetal. These characteristic signals indicate that PRPFc nanoparticles have been successfully synthesized. TEM results (Fig. 1D) show that both PRPFc and PRPFc@siNSUN2 nanoparticles are spherical in shape, suggesting that loading siNSUN2 does not alter the morphology of the PRPFc nanoparticles. Furthermore, in the presence of H_2_O_2_ (0.1 mM), the PRPFc component in PRPFc@siNSUN2 nanoparticles undergoes cleavage, generating fragments of various sizes. This is due to the breaking of the thioacetal structure under ROS stimulation, releasing ferrocene groups. The released ferrocene, due to its electron donor–acceptor-conjugated structure, further induces ROS generation, realizing a self-catalytic degradation “snowball” effect [25], thereby accelerating the release of siNSUN2. This phenomenon demonstrates the ROS responsiveness of PRPFc@siNSUN2. The in vitro drug release experiment results (Fig. 1E) show that under normal PBS, siNSUN2 in PRPFc@siNSUN2 nanoparticles is released slowly and steadily. When H_2_O_2_ is present, the ROS-responsive groups in PRPFc@siNSUN2 react specifically with H_2_O_2_, causing a change in the hydrophilic–hydrophobic properties of the polymer chain and disrupting its structure, which promotes rapid release of siNSUN2. About 72% of the drug is released within 12 h, further confirming the ROS responsiveness of PRPFc@siNSUN2 nanoparticles. The DLS results (Fig. 1F) show that the average particle sizes of PRPFc and PRPFc@siNSUN2 nanoparticles are 85.79 ± 0.67 nm and 88.79 ± 1.14 nm, respectively, with the encapsulation efficiency of siNSUN2 in PRPFc@siNSUN2 being 83.10% and the drug loading capacity being 13.85% (Table S1).

In addition, the stability of drug delivery carriers is an important prerequisite for their functionality. To investigate the stability of PRPFc@siNSUN2, its stability in a medium (H_2_O) was studied. The results showed that both PRPFc and PRPFc@siNSUN2 exhibited good stability in water and maintained this stability for at least 7 days (Fig. 1G). Further analysis using DLS was performed to investigate the changes in particle size and drug loading after storing PRPFc@siNSUN2 under different temperature conditions (4 °C, 25 °C, and 37 °C) for 30 days. The results (Fig. 1H and I) revealed that the changes in particle size and drug loading of PRPFc@siNSUN2 nanoparticles were temperature-dependent and time-dependent. At 4 °C, PRPFc@siNSUN2 nanoparticles maintained good colloidal stability and storage stability, with no significant changes in particle size or drug loading. However, under 25 °C and 37 °C conditions, the particle size gradually increased and drug loading decreased over time, indicating that low-temperature conditions are beneficial for maintaining its stability. This temperature dependence may be attributed to the impact of high temperatures on the aggregation behavior of nanoparticles and their drug loading capacity. According to the Stokes-Einstein equation, an increase in temperature and a decrease in dynamic viscosity lead to an increase in the diffusion constant. The higher the diffusion constant, the faster the particles diffuse. As the kinetic energy increases, the repulsive forces between particles become easier to overcome, resulting in particle aggregation [32]. In addition, the decrease in drug loading of PRPFc@siNSUN2 may be related to the poor thermal stability of its hydrophilic PEG chains. Previous studies have shown that PEG can enhance the dispersion of nanoparticles and prevent aggregation [33], but as temperature increases, its conformation may change [34], which could reduce its protective effect on siNSUN2, making it easier for siNSUN2 to diffuse out and thereby decreasing the drug loading. In conclusion, these results demonstrate that we have successfully prepared ROS-responsive PRPFc@siNSUN2 nanoparticles, which exhibit good morphology and stability.

### PRPFc@siNSUN2’s in vitro cytotoxicity, anti-GC efficacy, and AGS cell uptake

**Fig. 2.**
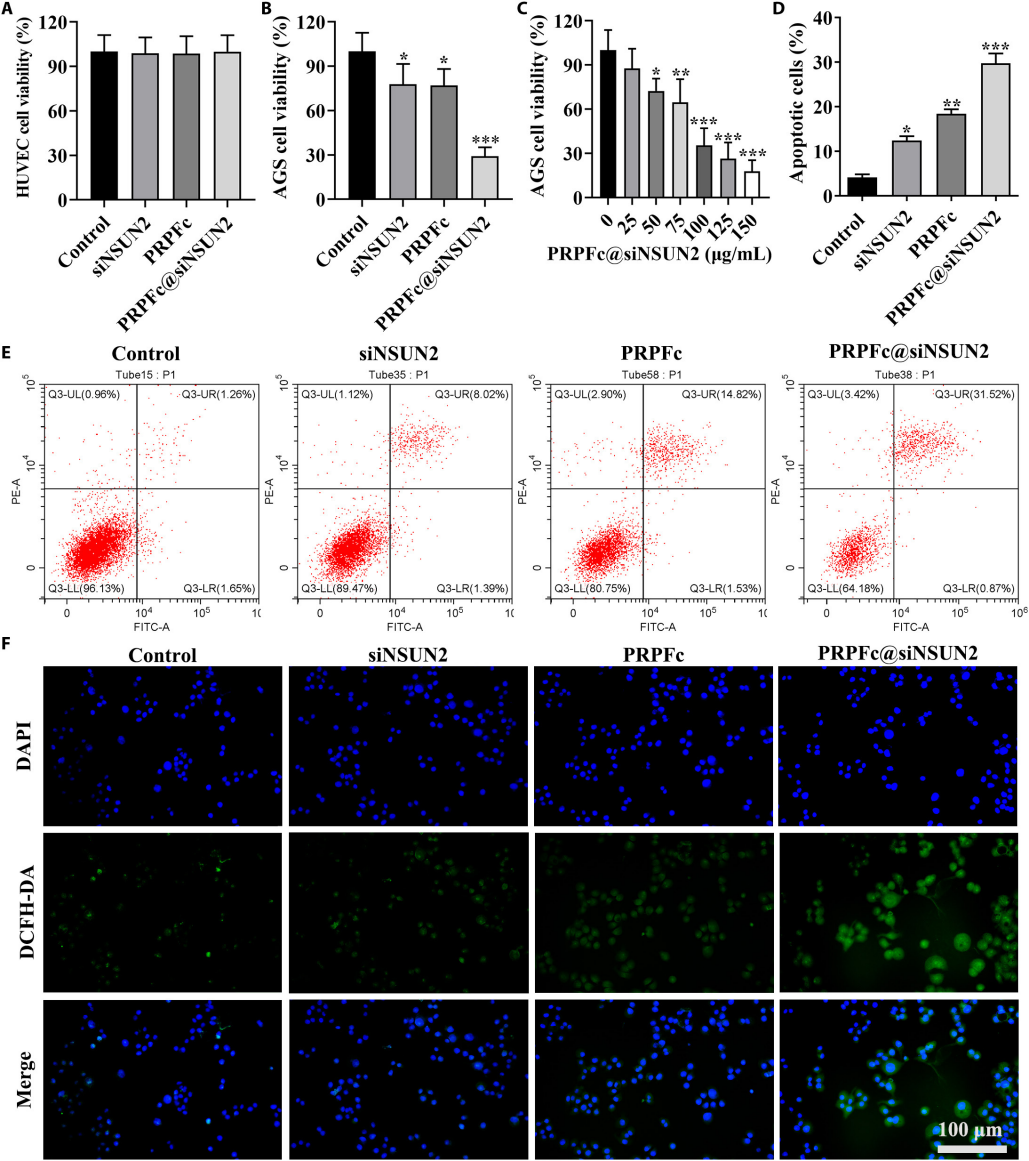
In vitro cytotoxicity and anticancer activity of PRPFc@siNSUN2. (A) Cytotoxicity of PRPFc@siNSUN2 on HUVECs. (B and C) Cytotoxicity of PRPFc@siNSUN2 on AGS cells. (D and E) Effects of PRPFc@siNSUN2 on apoptosis of AGS cells. (F) Fluorescence microscopy imaging of the impact of PRPFc@siNSUN2 on ROS levels in AGS cells, scale bars = 100 μm. ^*^P < 0.05, ^**^P < 0.01, ^***^P < 0.001 vs. Control.

An ideal drug delivery system should enhance the targeting of delivered drugs while minimizing toxicity to normal cells. Therefore, to assess the cytotoxicity of PRPFc and PRPFc@siNSUN2, we employed the CCK-8 assay to evaluate their effects on the viability of healthy HUVECs and GC AGS cells. The results demonstrated no significant impact on cell viability in HUVECs treated with siNSUN2, PRPFc, or PRPFc@siNSUN2 (Fig. 2A), indicating negligible cytotoxicity of PRPFc@siNSUN2 toward normal cells. In contrast, siNSUN2 significantly reduced AGS cell viability (*P* < 0.05). Loading the same dose of siNSUN2 into PRPFc resulted in enhanced inhibition of AGS cells, attributed to efficient nanoparticle uptake by cells [28] (*P* < 0.001) (Fig. 2B). Additionally, dose-dependent inhibition of AGS cell viability by PRPFc@siNSUN2 was observed, achieving over 50% inhibition at 100 μg/ml (Fig. 2C). These findings illustrate that PRPFc@siNSUN2 significantly enhances the inhibitory effect of siNSUN2 on AGS cell viability. Further apoptosis assays (Fig. 2D and E) revealed that compared to the control group, siNSUN2, PRPFc, and PRPFc@siNSUN2 significantly promoted apoptosis in AGS cells (*P* < 0.05), with PRPFc@siNSUN2 exerting a more pronounced pro-apoptotic effect (*P* < 0.001). This underscores the enhanced anti-GC efficacy of PRPFc@siNSUN2.

The levels of ROS in tumor tissues are approximately 10 times higher than those in normal tissues [35], making ROS an ideal marker for targeted drug delivery in cancer treatment. To investigate the impact of siNSUN2, PRPFc, and PRPFc@siNSUN2 on intracellular ROS levels, we utilized fluorescence microscopy imaging. The results (Fig. 2F) demonstrated that siNSUN2 reduced ROS production. However, the ROS levels in the PRPFc and PRPFc@siNSUN2 groups were higher than those in the siNSUN2 group. This difference is likely due to the ROS-responsive nature of the acyl-thioacetal moiety within the PRPFc@siNSUN2 nanoparticles [31]. Under high ROS conditions, PRPFc@siNSUN2 releases ferrocene from its structure, which undergoes further oxidation in the presence of ROS, inducing additional ROS production. This positive feedback mechanism triggers a rapid increase in ROS levels [36], overwhelming the antioxidant defense system and pushing the cells into oxidative stress. As a result, cellular components such as lipids, proteins, and DNA are damaged [37]. Persistent oxidative stress is a hallmark of cancer cells and serves as the foundation for developing new therapeutic strategies based on ROS inducers [38]. Additionally, studies have shown that ferrocene and its derivatives exhibit promising potential in such treatments [39]. In conclusion, these findings suggest that PRPFc@siNSUN2 nanoparticles exert anti-GC effects by elevating ROS levels and inducing oxidative stress in cancer cells.

**Fig. 3.**
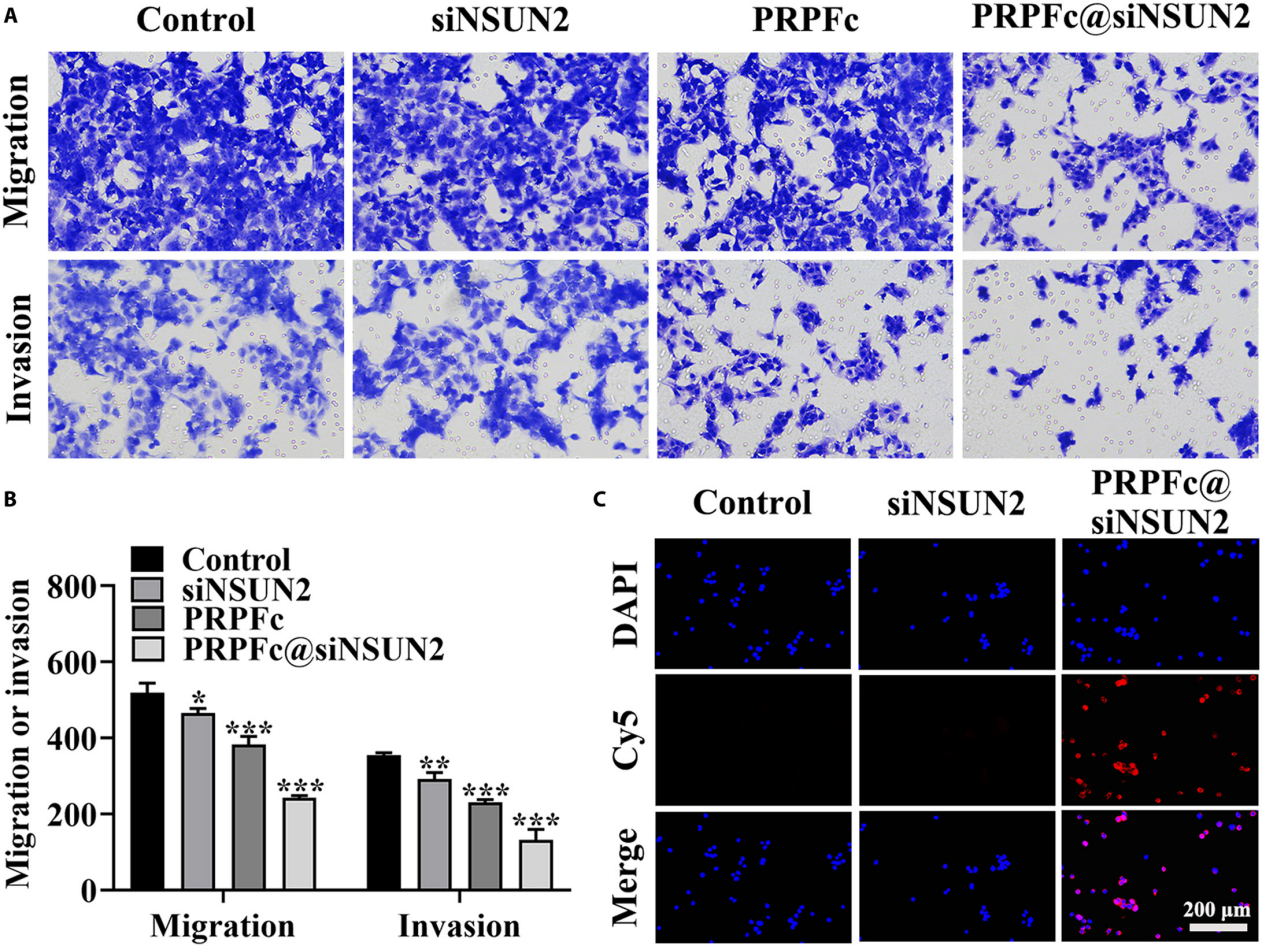
Effects of PRPFc@siNSUN2 on migration, invasion, and uptake of AGS cells. (A and B) Influence of PRPFc@siNSUN2 on the migration and invasion of AGS cells. (C) Impact of PRPFc@siNSUN2 on the uptake of AGS cells, scale bars = 200 μm. ^*^P < 0.05, ^**^P < 0.01, ^***^P < 0.001 vs. Control.

Metastasis is the primary cause of treatment failure in cancer, making the inhibition of tumor cell migration a crucial aspect of cancer therapy. To investigate the effect of PRPFc@siNSUN2 on the migration and invasion of GC cells, we conducted Transwell assays to assess AGS cell migration and invasion capabilities under different interventions. The results (Fig. 3A and B) show that compared to the control group, siNSUN2, PRPFc, and PRPFc@siNSUN2 significantly reduced cell migration and invasion (*P* < 0.05), with PRPFc@siNSUN2 exhibiting the most significant decrease (*P* < 0.001). This indicates that PRPFc@siNSUN2 significantly enhances the antimigration and invasion effects of siNSUN2 on cells. Notably, PRPFc nanoparticles without loaded siNSUN2 also exhibited anti-AGS cell effects, including inhibition of cell viability, migration, and invasion, as well as promotion of apoptosis. This effect may be attributed to the ability of ferrocene within the PRPFc nanoparticles to catalyze the conversion of H_2_O_2_ into •OH, which kills AGS cells and thereby exerts an antitumor effect [40].

**Fig. 4.**
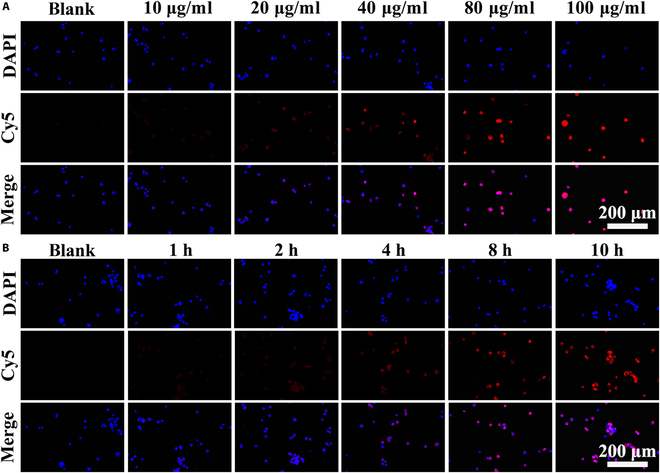
Effects of PRPFc@siNSUN2 on the cellular uptake of AGS cells. (A) Influence of PRPFc@siNSUN2 at different concentrations on the cellular uptake by AGS cells. (B) Impact of PRPFc@siNSUN2 at different exposure times on the cellular uptake by AGS cells.

One of the key prerequisites for efficient gene silencing by siRNA is its high cellular uptake rate, and effective intracellular delivery is essential for therapeutic success [41]. Thus, we investigated the uptake of siNSUN2. As shown in Fig. 3C, the amount of PRPFc@siNSUN2 internalized by AGS cells was significantly higher than that of free siNSUN2. The poor uptake of free siNSUN2 can be attributed to its high molecular weight and negative charge [42], which hinder cellular entry. The enhanced uptake may be associated with the PEG component in the PRPFc structure. PEG not only exhibits “stealth” properties by reducing protein adsorption but also mitigates phagocytosis by the reticuloendothelial system [43]. Numerous studies have demonstrated that PEGylated nanoparticles effectively deliver therapeutic agents and enhance cellular uptake [44]. In this study, the improved uptake of PRPFc@siNSUN2 may be due to the presence of PEG in the PRPFc structure. Additionally, further experiments revealed that AGS cell uptake of PRPFc@siNSUN2 was time- and concentration-dependent (Fig. 4). These results suggest that PRPFc@siNSUN2 promotes siNSUN2 uptake, thereby enhancing its ability to regulate AGS cell viability, apoptosis, migration, and invasion.

### PRPFc@siNSUN2 exhibits excellent tumor-targeting capability

**Fig. 5.**
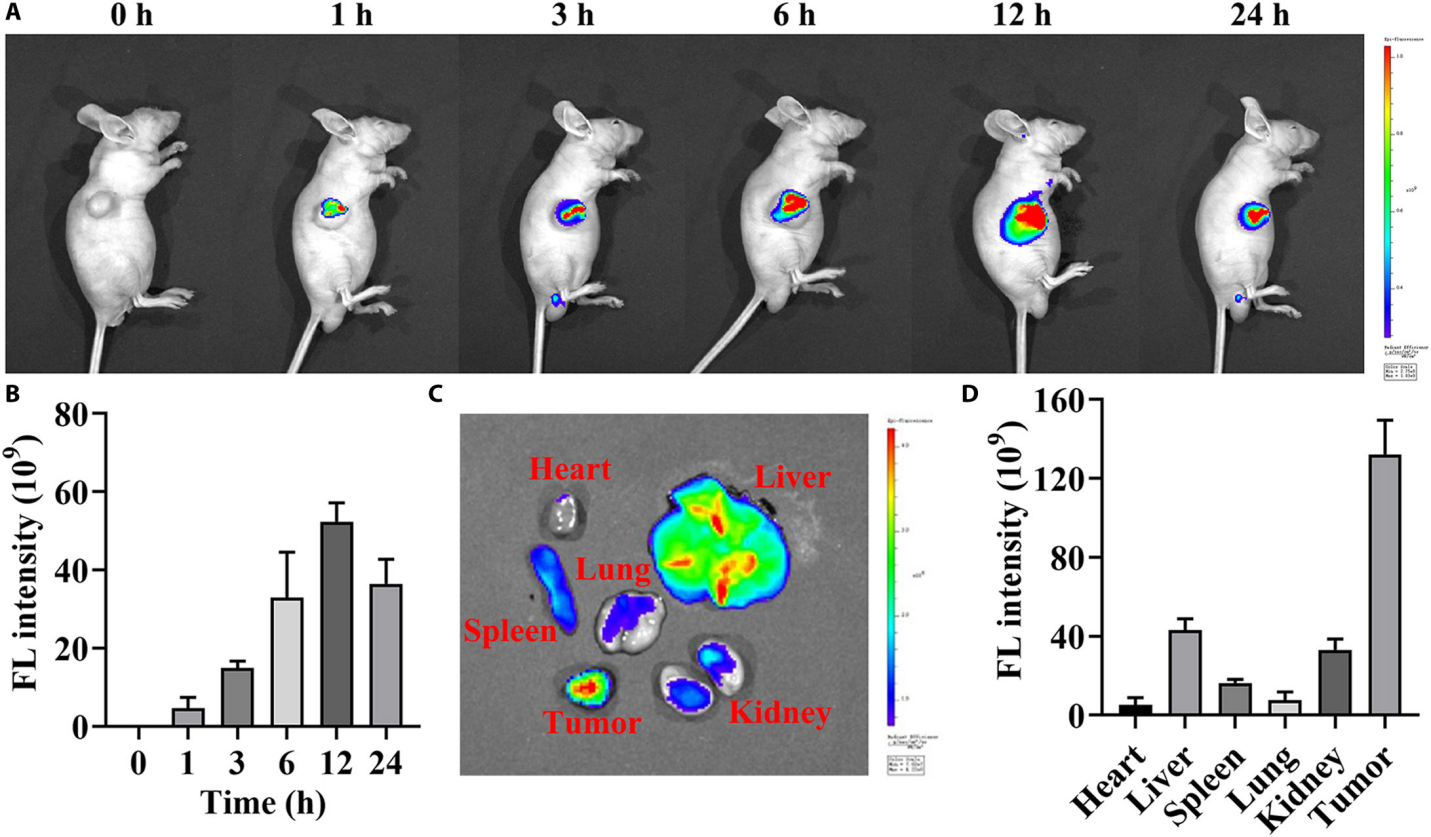
Tumor targeting capability of PRPFc@siNSUN2. (A and B) In vivo fluorescence imaging analysis. (C and D) Ex vivo fluorescence imaging analysis of tissues and major organs.

To further investigate the biodistribution of PRPFc@siNSUN2, PRPFc@siNSUN2-Cy5 was injected into AGS tumor-bearing mice via tail vein for in vivo imaging analysis. Fluorescence imaging results in mice revealed (Fig. 5A and B) a significant fluorescence signal at the tumor site, reaching its peak at 12 h and gradually decreasing thereafter, indicating the excellent tumor-targeting capability of PRPFc@siNSUN2. Further ex vivo fluorescence imaging of the tumor and major organs (Fig. 5C and D) showed the strongest fluorescence signal in the tumor tissue, which may be attributed to the structural disruption of PRPFc in the ROS-rich tumor microenvironment, facilitating nanoparticle degradation. The degradation of nanoparticles results in the release of siNSUN2-Cy5, which contributes to enhanced fluorescence to some extent [45]. In conclusion, these findings demonstrate the strong tumor-targeting ability of PRPFc@siNSUN2.

### In vivo anti-GC efficacy of PRPFc@siNSUN2

**Fig. 6.**
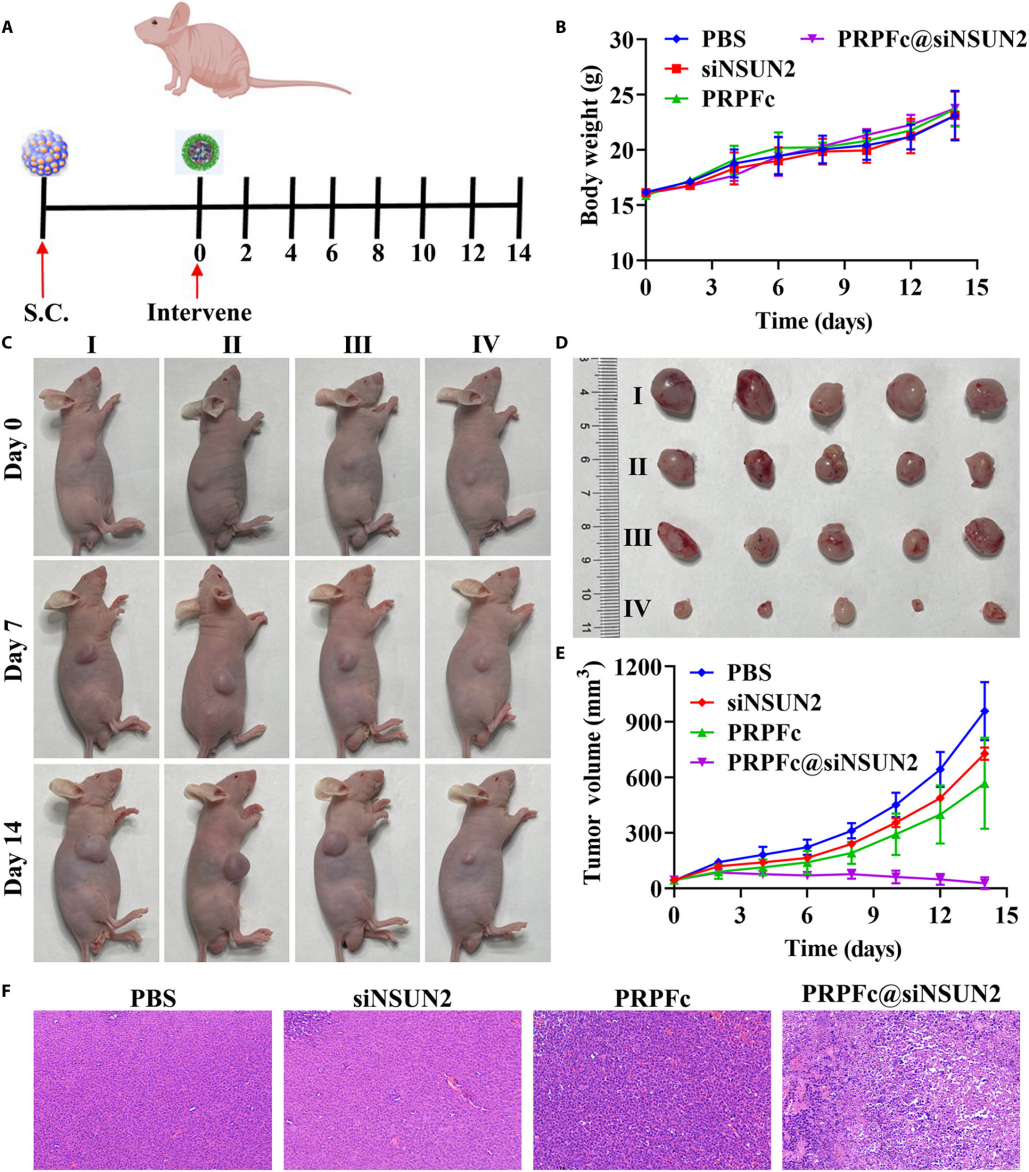
In vivo anti-gastric cancer activity of PRPFc@siNSUN2. (A) Treatment protocol of PRPFc@siNSUN2, involving the injection of AGS cells into the right dorsal region of BALB/c nude mice to establish orthotopic tumors. When the tumor size reached approximately 50 mm^3^, the mice were divided into different groups and received respective intervention treatments. The mice were euthanized on the 14th day. (B) Changes in body weight of mice in each group. (C) Images of mice in each group. (D) Images of tumor tissues in each group. (E) Changes in tumor volume in each group. (F) H&E-stained images of tumor tissues in each group. I, PBS. II, siNSUN2. III, PRPFc. IV, PRPFc@siNSUN2.

Based on our preliminary experimental findings, we have confirmed the enhanced anti-GC activity and in vivo tumor targeting capability of PRPFc@siNSUN2. To further investigate the in vivo anti-GC efficacy of PRPFc@siNSUN2, we established an AGS tumor mouse model and compared the therapeutic effects of different interventions (Fig. 6A). The results demonstrated (Fig. 6C to E) that compared to the PBS group, the tumor volumes were reduced in the siNSUN2 group, PRPFc group, and PRPFc@siNSUN2 group. Notably, the inhibitory effect of PRPFc@siNSUN2 on tumor volume was superior to that of the siNSUN2 group and PRPFc group, indicating the enhanced tumor-suppressive effect of PRPFc@siNSUN2 in combination with siNSUN2. Further histological examination of tumor tissues using H&E staining (Fig. 6F) revealed no significant cell necrosis in the tumor tissues of the PBS group, while cell necrosis and inflammatory changes were observed in the other 3 groups. Among them, the effect of PRPFc@siNSUN2 was the most significant, consistent with the inhibitory effect observed in the in vitro experiments on AGS cells. Importantly, compared to the PBS group, no significant impact on the body weight of mice was observed in the siNSUN2, PRPFc, and PRPFc@siNSUN2 groups, indicating the absence of obvious systemic toxicity of PRPFc and PRPFc@siNSUN2 in the AGS tumor mouse model (Fig. 6B). Although existing literature has reported the application of ferrocene-based ROS-responsive nanocarriers [28,40] or other types of ROS-responsive nanocarriers [46] in anticancer therapy, there have been no reports to date on their use for the delivery of siNSUN2. In this study, by constructing PRPFc@siNSUN2 nanoparticles, we achieved the stable loading of siNSUN2 and its triggered release in high ROS environments, demonstrating superior anticancer effects in both GC cells and animal models. Moreover, the main challenge in traditional siRNA therapy for GC lies in the difficulty of accurately delivering siRNA to targeted cancer cells in vivo. Unmodified siRNA has many limitations in terms of stability, cellular uptake, and off-target effects [47]. In this study, PRPFc@siNSUN2 utilizes the high levels of ROS in the GC tumor microenvironment to induce its structural cleavage, releasing the encapsulated siNSUN2, successfully achieving the targeted release of siNSUN2. The results further demonstrate that, compared to free siNSUN2, PRPFc@siNSUN2 nanoparticles significantly enhance the anti-GC efficacy of siNSUN2, exhibit better stability, and effectively improve the uptake of siNSUN2 by GC cells. In conclusion, these results suggest that PRPFc@siNSUN2 enhances the anti-GC efficacy of siNSUN2 and provides a new strategy for precision treatment of GC in the future.

### In vivo biosafety evaluation

**Fig. 7.**
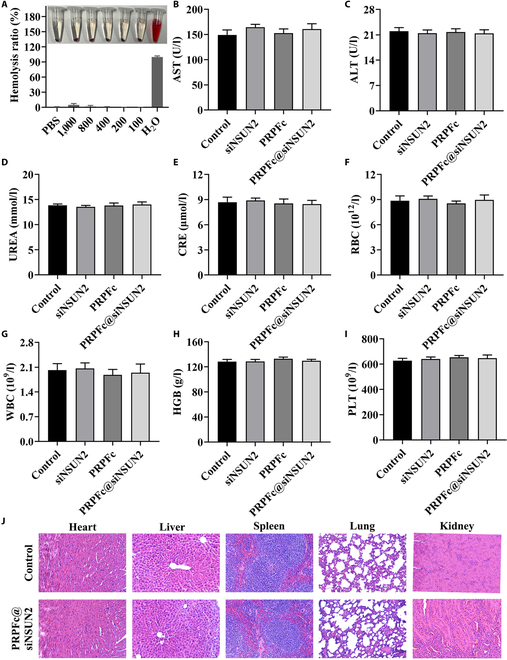
Biocompatibility analysis. (A) Hemocompatibility assessment. (B to I) Quantification of AST, ALT, UREA, CRE, RBC, WBC, HGB, and PLT levels in serum using an automated biochemical analyzer. (J) Histological examination of heart, liver, spleen, lung, and kidney tissues using H&E staining in both groups of rats. AST, aspartate aminotransferase; ALT, alanine aminotransferase; UREA, urea; CRE, creatinine; RBC, red blood cells; WBC, white blood cells; HGB, hemoglobin; PLT, platelets.

In vivo biosafety evaluation is crucial for assessing the clinical feasibility of nanomedicines. Therefore, we conducted a thorough investigation of the biocompatibility of PRPFc@siNSUN2. Although nanoparticles offer advantages in size and surface properties, intravenous injection can potentially cause significant hemolysis, posing safety risks. Evaluating hemolytic activity is therefore essential to avoid severe side effects during in vivo administration. In this study, the hemolysis assay revealed that even at a concentration of 1,000 μg/ml, PRPFc@siNSUN2 exhibited a hemolysis rate of less than 3% (Fig. 7A). According to the American Society for Testing and Materials (ASTM) standards, a hemolysis rate below 5% is considered indicative of good hemocompatibility [48]. These results demonstrate that PRPFc@siNSUN2 is nonhemolytic and suitable for applications requiring blood compatibility in drug delivery systems. Further hematological analyses (Fig. 7B to I) indicated no significant differences in the levels of ALT, AST, UREA, CRE, RBC, WBC, HGB, and PLT among the treatment groups. Additionally, histological examination of major organs, including the heart, liver, spleen, lungs, and kidneys, showed no significant pathological changes, inflammation, or abnormalities in the PRPFc@siNSUN2-treated group (Fig. 7J). These findings suggest that PRPFc@siNSUN2 does not induce organ toxicity. Moreover, prior studies have demonstrated that ferrocene-containing stimuli-responsive polymers can yield aqueous nanoparticles with enhanced biocompatibility [49]. Collectively, these results confirm that PRPFc@siNSUN2 exhibits excellent biocompatibility and holds significant potential for clinical applications.

## Conclusion

**Fig. 8.**
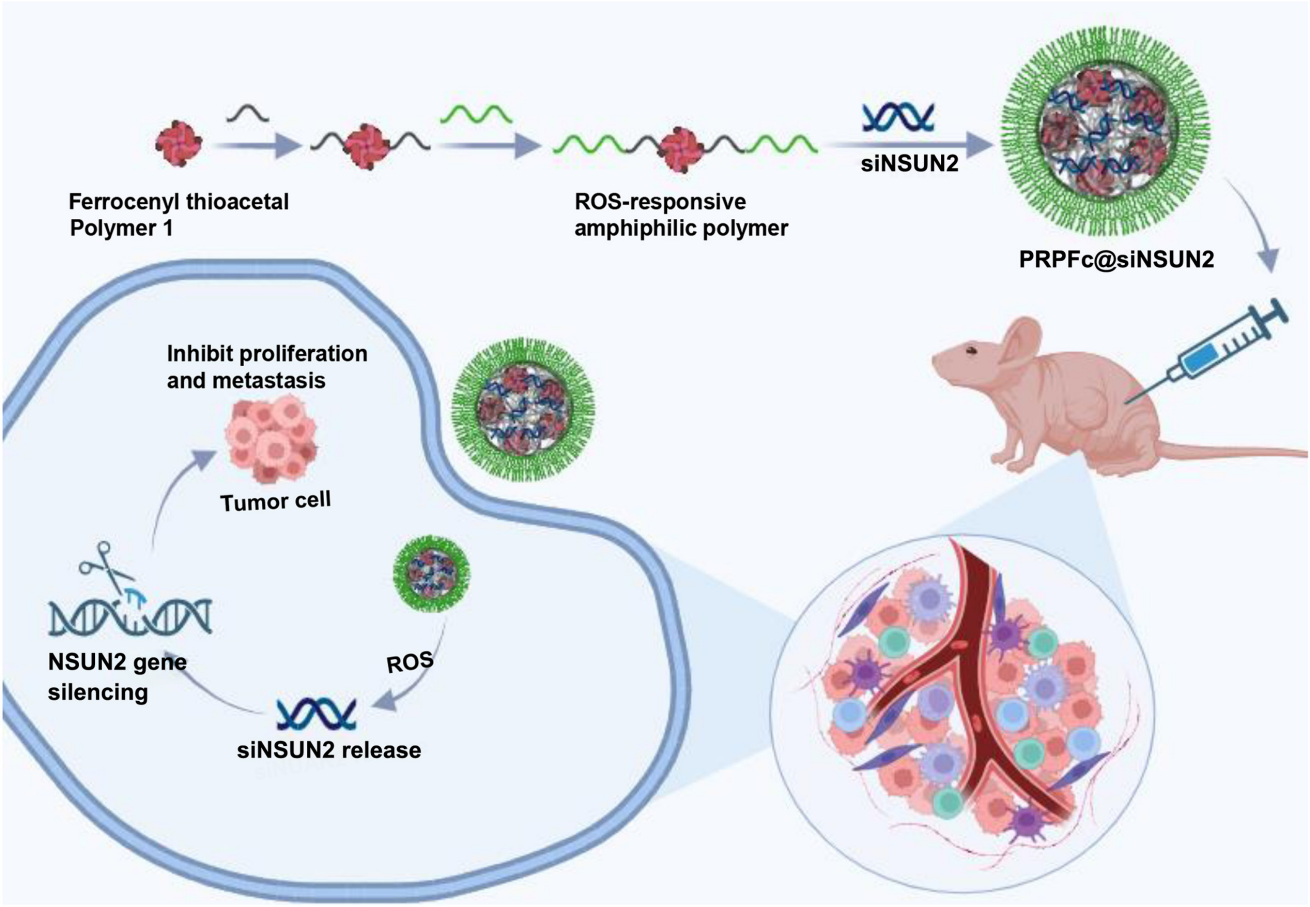
PRPFc@siNSUN2 inhibits the progression of gastric cancer in vitro and in vivo.

To the best of our knowledge, this study successfully developed ROS-responsive ferrocene nanoparticles (PRPFc@siNSUN2) loaded with siNSUN2 for the first time. PRPFc@siNSUN2 nanoparticles are spherical in shape, with an average particle size of 88.79 ± 1.14 nm, encapsulation efficiency of 83.10%, and drug loading of 13.85%. They exhibit good colloidal stability and storage stability. In an H_2_O_2_ environment, PRPFc@siNSUN2 nanoparticles can undergo structural cleavage and release siNSUN2. The results of this study show that these nanoparticles have low cytotoxicity and good biocompatibility, confirming their potential clinical application value. In vitro experiments demonstrated that PRPFc@siNSUN2 nanoparticles enhance the therapeutic effect of siNSUN2 on GC. Compared to free siNSUN2, these nanoparticles significantly inhibit cell proliferation, migration, and invasion in GC cells, promote apoptosis, and increase intracellular ROS levels. Moreover, the cellular uptake efficiency of PRPFc@siNSUN2 was significantly improved, effectively inhibiting GC cell growth. In vivo experiments further validated the superiority of PRPFc@siNSUN2 in GC suppression, with its antitumor effect significantly outperforming free siNSUN2 and showing good tumor targeting (Fig. 8). In conclusion, the results of this study indicate that PRPFc@siNSUN2 not only significantly enhances the therapeutic effect of siNSUN2 but also provides a novel, safe, and effective strategy for the treatment of GC. These findings lay the foundation for future targeted therapy research in GC.

## Ethical Approval

All animal experimental procedures in this study complied with the guidelines for the care and use of laboratory animals. The research protocol was approved by the Laboratory Animal Ethics Committee of Wenzhou Medical University (approval no. wydw2024-0323).

## Data Availability

All data can be provided as needed.
