# Supplementary material for: Reactive Oxygen Species-Responsive Ferrocene Nanoparticles Delivering Small Interfering RNA Targeting NOP2/Sun RNA Methyltransferase Family Member 2 for Gastric Cancer Therapy
Source: Biomater Res. 2025 May 29;29:0209. doi: 10.34133/bmr.0209 (PMC12120247; doi:10.34133/bmr.0209)
Supplement: Supplementary 1 — Table S1 Fig. S1 [file bmr.0209.f1.zip › Supplementary Figure 1.docx]

**Supplementary Figure 1** Synthesis of ROS responsive amphiphilic polymer PRPFc.
